# Development of prognosis model for colon cancer based on autophagy-related genes

**DOI:** 10.1186/s12957-020-02061-w

**Published:** 2020-10-30

**Authors:** Xu Wang, Yuanmin Xu, Ting Li, Bo Chen, Wenqi Yang

**Affiliations:** Department of General Surgery, The First Affiliated Hospital of Anhui Medical University, Hefei, 230032 Anhui China

**Keywords:** Autophagy-related genes, Prognosis model, Colon cancer, TCGA

## Abstract

**Background:**

Autophagy is an orderly catabolic process for degrading and removing unnecessary or dysfunctional cellular components such as proteins and organelles. Although autophagy is known to play an important role in various types of cancer, the effects of autophagy-related genes (ARGs) on colon cancer have not been well studied.

**Methods:**

Expression profiles from ARGs in 457 colon cancer patients were retrieved from the TCGA database (https://portal.gdc.cancer.gov). Differentially expressed ARGs and ARGs related to overall patient survival were identified. Cox proportional-hazard models were used to investigate the association between ARG expression profiles and patient prognosis.

**Results:**

Twenty ARGs were significantly associated with the overall survival of colon cancer patients. Five of these ARGs had a mutation rate ≥ 3%. Patients were divided into high-risk and low-risk groups based on Cox regression analysis of 8 ARGs. Low-risk patients had a significantly longer survival time than high-risk patients (*p* < 0.001). Univariate and multivariate Cox regression analysis showed that the resulting risk score, which was associated with infiltration depth and metastasis, could be an independent predictor of patient survival. A nomogram was established to predict 1-, 3-, and 5-year survival of colon cancer patients based on 5 independent prognosis factors, including the risk score. The prognostic nomogram with online webserver was more effective and convenient to provide information for researchers and clinicians.

**Conclusion:**

The 8 ARGs can be used to predict the prognosis of patients and provide information for their individualized treatment.

**Supplementary Information:**

The online version contains supplementary material available at 10.1186/s12957-020-02061-w.

## Introduction

Despite rapid advances in medical science and technology, cancer incidence and cancer-related mortality rates are increasing rapidly worldwide [1]. Patients with early stage colon cancer can be successfully treated by surgery; however, most patients with advanced colon cancer experience recurrence and metastasis and typically exhibit 5-year survival rates < 10% [2–4]. Although tumor size, stage, and histological grade are often used to predict prognosis of colon cancer patients, these indicators do not accurately predict patient survival and are not useful for developing individualized treatment regimens. With the development of chemotherapy and targeted therapeutics, the overall survival rate of colon cancer patients has increased significantly. Carcinoembryonic antigen (CEA) has been widely used in colon cancer diagnosis, but more efficient molecular biomarkers for early diagnosis and advanced therapeutic agents are needed to improve prognosis and treatment outcomes in colon cancer patients [5].

Autophagy is a multi-step process of intracellular degradation closely controlled by numerous ARGs, which occurs under a variety of stress conditions, including organelle damage, the presence of abnormal proteins, and nutritional deficiency [6]. Autophagy plays an important role in various aspects of tumor suppression, including the response of cells to nutrition and hypoxia stress, control of programmed cell death, and tumor-related immune response. Under normal physiological conditions, autophagy keeps cells in a stable state, prevents the accumulation of damaged and potentially carcinogenic proteins and organelles, and inhibits the process of carcinogenesis. However, once tumors begin to form, autophagy provides an abundance of nutrients for cancer cells and promotes tumor growth [7].

Over the past two decades, abundant researches have provided important information on the correlation between autophagy and colon cancer [8–10]. For example, Schroll et al. [9] suggested that cancer cells may become more sensitive to chemotherapy in an environment of glucose restriction and autophagy inhibition. Autophagy is now widely recognized to play an important role in colon cancer growth and progression and may be useful in anti-cancer therapies [11]. Previous studies have focused primarily on relationships between one or several ARGs and colon cancer, but limited research has been devoted to large scale searches for ARGs related to patient prognosis. In this study, we contribute to this growing area of research by exploring the value of ARGs in predicting prognosis of colon cancer patients and improving clinical decision-making for individualized treatment. We used clinical data and large-scale patterns of ARG expression in colon cancer patients to develop an informative model of prognosis.

## Materials and methods

### ARG identification and expression

A list of 232 human ARGs was constructed using the Human Autophagy Database (HADb; http://www.autophagy.lu), a publicly available repository containing up-to-date information on human genes and proteins that are directly or indirectly involved in autophagy. Expression patterns for the 232 ARGs and clinical information from 457 colon cancer patients were downloaded from the Genomic Data Commons (GDC) Data Portal (https://portal.gdc.cancer.gov).

### GO and KEGG analysis

To better understand the biological functions of the ARGs, gene oncology (GO) and Kyoto encyclopedia of genes and genomes (KEGG) analyses were performed using “ggplot2,” “Bioconductor,” and “org.Hs.eg.db” R packages. GO and KEGG pathways with *p* values and *q* values < 0.05 were considered to be significant.

### Establishment of an ARG-related prognosis model

Twenty ARGs that were significantly related to patient prognosis (*p* < 0.05) were identified using univariate Cox regression analysis. The cBioPortal for Cancer Genomics online website (https://www.cbioportal.org) was used to determine the mutation rate for each of the 20 ARGs. A risk score for each patient was calculated based on the expression of these ARGs using multivariate Cox regression analysis. Patients were then divided into high-risk and low-risk groups based on the risk score; the median risk score was used to differentiate the two groups. The Kaplan-Meier method was used to evaluate survival differences between the high- and low-risk groups, and the log-rank statistical method was used for comparison. Univariate and multivariate analyses were used to determine if the risk score was an independent predictor of prognosis in colon cancer patients. Receiver operator characteristic curves (ROC) and area under the curves (AUC) were used to test the prediction efficiency of the prognosis model. A nomogram was established based on five independent prognosis factors that were significant in both the univariate and multivariate analyses (*p* < 0.05). Calibration graphs were drawn to show the differences between nomogram-predicted and actual survival rates of the colon cancer patients. Online version of the nomogram was established using “DynNom” and “shiny” R packages and deployed using shinyapps online website.

### Statistical analysis

All statistical analyses were conducted using R programming language (v.4.0.2). Results with a *p* value < 0.05 were considered to be significant. Univariate Cox regression analysis was used to identify prognosis-related ARGs. Univariate and multivariate analyses were performed using the Cox proportional hazard model to identify factors that were independently related to prognosis of colon cancer patients. Survival curves were drawn using the Kaplan-Meier method and compared by a log-rank test.

## Results

### Differentially expressed ARGs

**Fig. 1:**
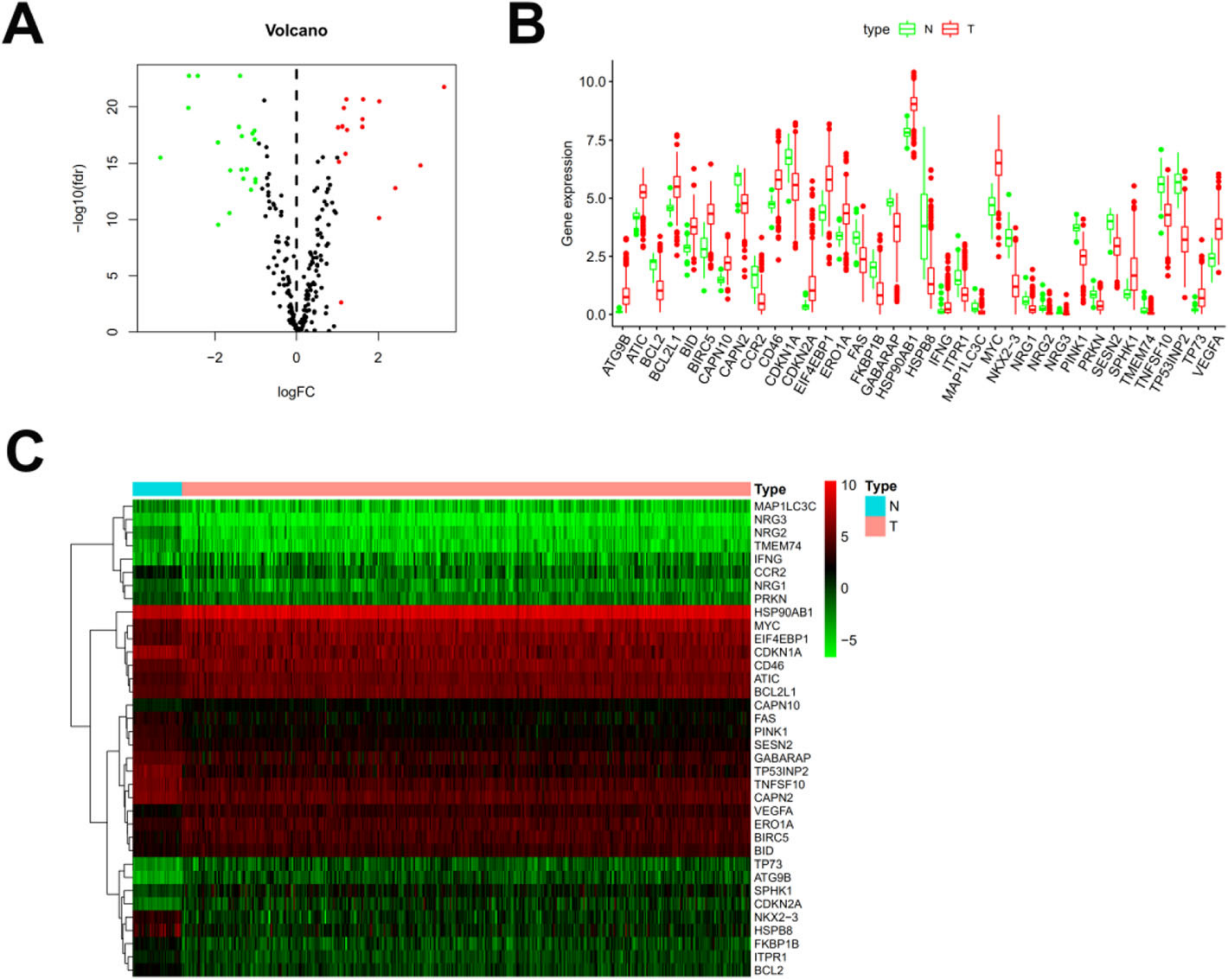
Differentially expressed autophagy-related genes. Heat map (**a**) and volcano map (**b**) show differentially expressed genes between colon tumor and normal tissues, with red dots representing significantly upregulated genes, green dots representing significantly downregulated genes, and black dots representing no differences gene. **c** Expression patterns of 36 autophagy-related genes (ARGs) in colon cancer types and paired non-tumor samples. Each red box plot represents a different tumor sample and green represents a non-tumor sample

**Fig. 2:**
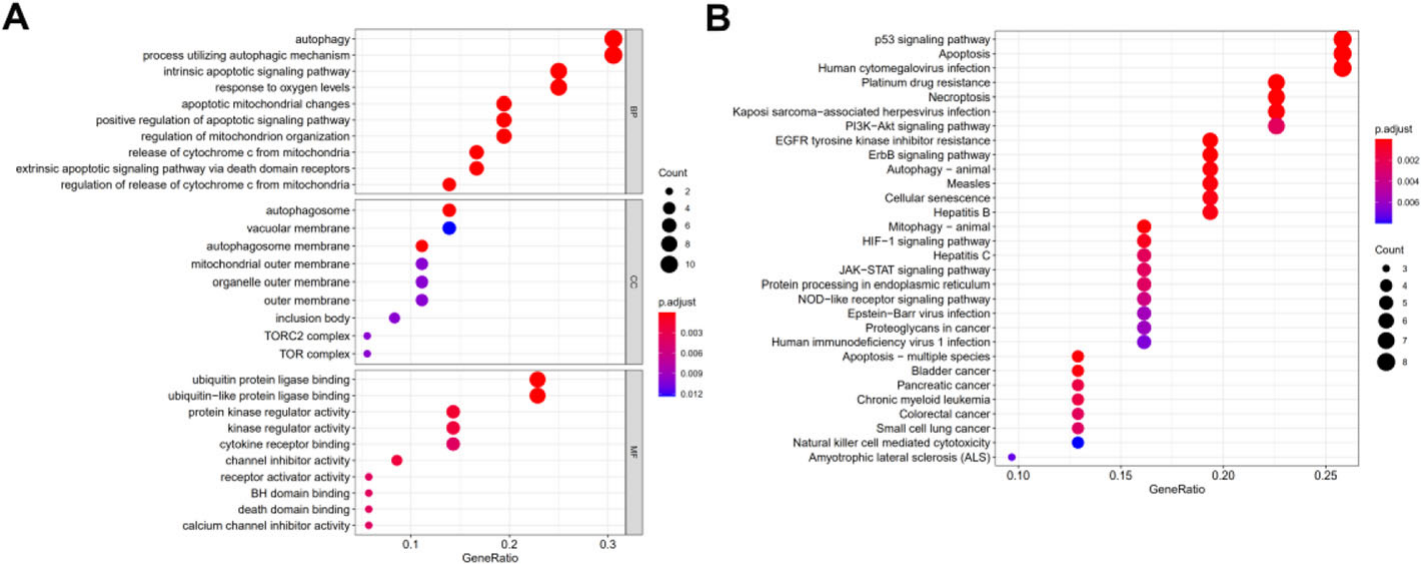
Gene functional enrichment of differentially expressed ARGs. **a** GO analysis shows the biological processes, cellular components, and molecular functions involved in differential genes. **b** KEGG shows the signaling pathway involved in differential ARGs

As shown in Fig. 1, 36 differentially expressed ARGs with a false discovery rate (FDR) < 0.05 and |logFC| > 1 were identified from 232 ARGs. A volcano map (Fig. 1a), boxplots (Fig. 1b), and a heatmap (Fig. 1c) indicated that 20 ARGs (BCL2, CAPN2, CCR2, CDKN1A, FAS, FKBP1B, GABARAP, HSPB8, ITPR1, MAP1LC3C, NKX2-3, NRG1, NRG2, NRG3, PINK1, PRKN, SESN2, TMEM74, TNFSF10, and TP53INP2) were downregulated while 16 ARGs (ATG9B, ATIC, BCL2L1, BID, BIRC5, CAPN10, CD46, CDKN2A, EIF4EBP1, ERO1A, HSP90AB1, IFNG, MYC, SPHK1, TP73, and VEGFA) were overexpressed in colon tumor tissues. Functional enrichment analysis identified numerous GO and KEGG enrichment pathways (Fig. 2). The 36 genes were primarily related to the molecular functions of autophagy, process utilizing autophagic mechanism, and intrinsic apoptotic signaling pathways. As seen in Fig. 2a, these 36 genes are mainly related to molecular functions (MF) of autophagy, process utilizing autophagic mechanism, and intrinsic apoptotic signaling pathway, they are correlated with cellular components (CC) of autophagosome, vacuolar membrane, and autophagosome membrane, the genes are also involved in biological processes (BP) of ubiquitin-protein ligase binding, ubiquitin-like protein ligase binding, and protein kinase regulator activity. These ARGs participate in the pathways of p53 signaling pathway, apoptosis, and human cytomegalovirus infection (Fig. 2b).

### Prognosis-related ARGs

**Fig. 3:**
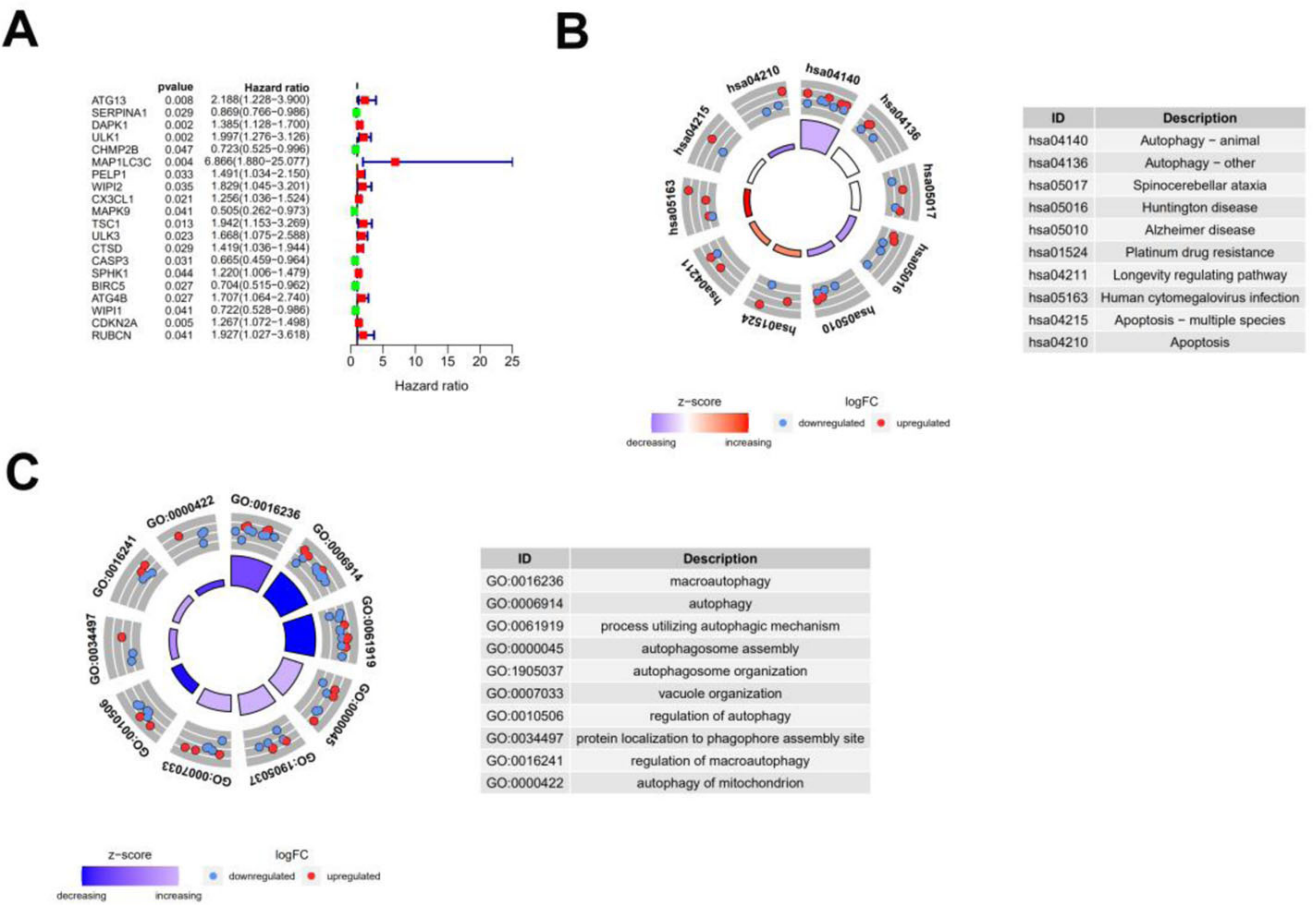
Expression profile and prognostic value of ARGs. **a** Risk ratio forest plot showed the prognostic value of the gene. **b** KEGG shows the signaling pathways involved in 20 prognostic-related ARGs. **c** GO analysis revealed the biological processes, cellular components, and molecular functions involved in 20 prognostic-related ARGs

**Fig. 4:**
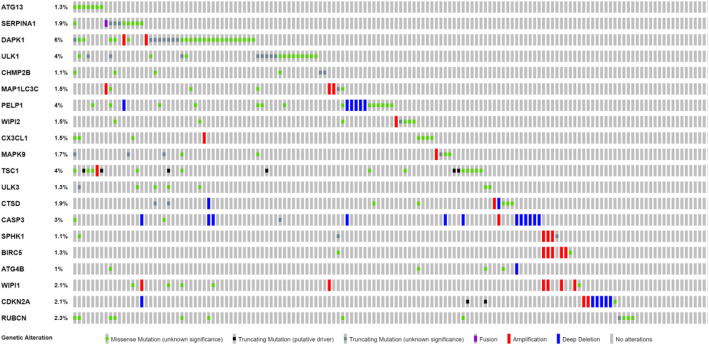
Mutations in prognosis-related ARGs. DAPK1 is the most frequently mutated gene. A total of 5 genes have a mutation rate ≥ 3%

A forest map identified 20 ARGs that were associated with prognosis in colon cancer patients (Fig. 3a). Of these 20 prognosis-related genes, six genes were determined to be protective and 14 ARGs were associated with increased risk. Results of the KEGG analysis indicated that prognostic ARGs were mainly involved in pathways of autophagy, spinocerebellar ataxia, and Huntington’s disease (Fig. 3b). Prognosis-related genes were correlated with macro-autophagy, autophagy, and process utilizing autophagic mechanism (Fig. 3c). Mutations in these 20 genes, examined using the cBioPortal website, showed that missense mutations, amplifications, and deep detection were the most common mutation types (Fig. 4). Five ARGs (DAPK1, ULK1, PELP1, TSC1, and CASP3) had a mutation rate ≥ 3%, among which DAPK1 had the highest mutation rate.

### Development of a prognosis model

**Fig. 5:**
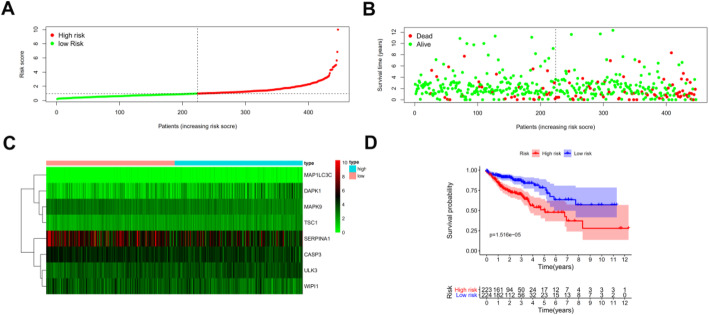
Development of a prognostic index based on ARGs. **a** Distribution of prognostic index. **b** Survival status of patients in different groups. **c** Heat map of the expression profile of the included ARGs. **d** Patients in the high-risk group have a significant shorter overall survival rate

Using multivariate analysis to develop a risk score for colon cancer patients, 8 ARGs were significantly related to prognosis. The risk score was defined as [Expression level of SERPINA1 × (−0.11979)] + [Expression level of DAPK1 × (−0.29697)] + [Expression level of MAP1LC3C × (1.50543)] + [Expression level of MAPK9 × (−0.62080)] + [Expression level of TSC1 × (−0.64199)] + [Expression level of ULK3 × (−0.31259)] + [Expression level of CASP3 × (−0.44136)] + [Expression level of WIPI1 × (−0.27200)]. Based on the risk score, patients were divided into high-risk and low-risk groups using the median risk score as the cut-off point between groups (Fig. 5a). Patients with higher risk scores were more likely to be deceased (Fig. 5b). A heatmap was used to show differences in expression for these 8 prognosis-related ARGs between groups (Fig. 5c).

**Fig. 6:**
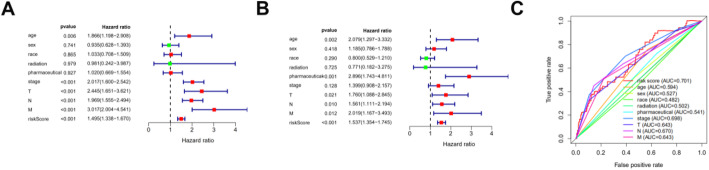
Prognostic model based on ARGs shows good predictive performance. A forest plot of univariate (**a**) and multivariate (**b**) Cox regression analysis in colon cancer. (**c**) Survival-dependent receiver operating characteristic (ROC) curves validate the prognostic significance of ARGs-based prognostic indicators

Clinicopathologic characteristics of TCGA colon cancer patients were downloaded from TCGA database (Additional Table 1). Examination of the survival curves for the low-risk and high-risk patient groups, drawn using the Kaplan-Meier method (Fig. 5d), showed that high-risk patients had a significantly lower probability of survival (*p* < 0.01). Univariate and multivariate analyses were performed to identify prognosis-related factors in colon cancer patients (Fig. 6a and b). Factors with a *p* value < 0.05 in the univariate analysis were included in the multivariate analysis. Forest maps showed that age, pharmaceutical use, tumor invasion depth, lymph node metastasis, distant metastasis, and the risk score were still significant after multivariate analysis. Therefore, the risk score was independently associated with prognosis of patients [Hazard ratio (HR) = 1.537, 95% CI = 1.354-1.745, *p* < 0.001; Fig. 6b]. AUC of the ROC were used to test the prediction efficiency of the prognosis model (Fig. 6c). AUC of the risk score (0.701) was greater than that for any other clinicopathologic characteristics, including the American Joint Committee on Cancer stage, which showed that the risk score could be a reliable predictor of prognosis in colon cancer patients.

**Fig. 7:**
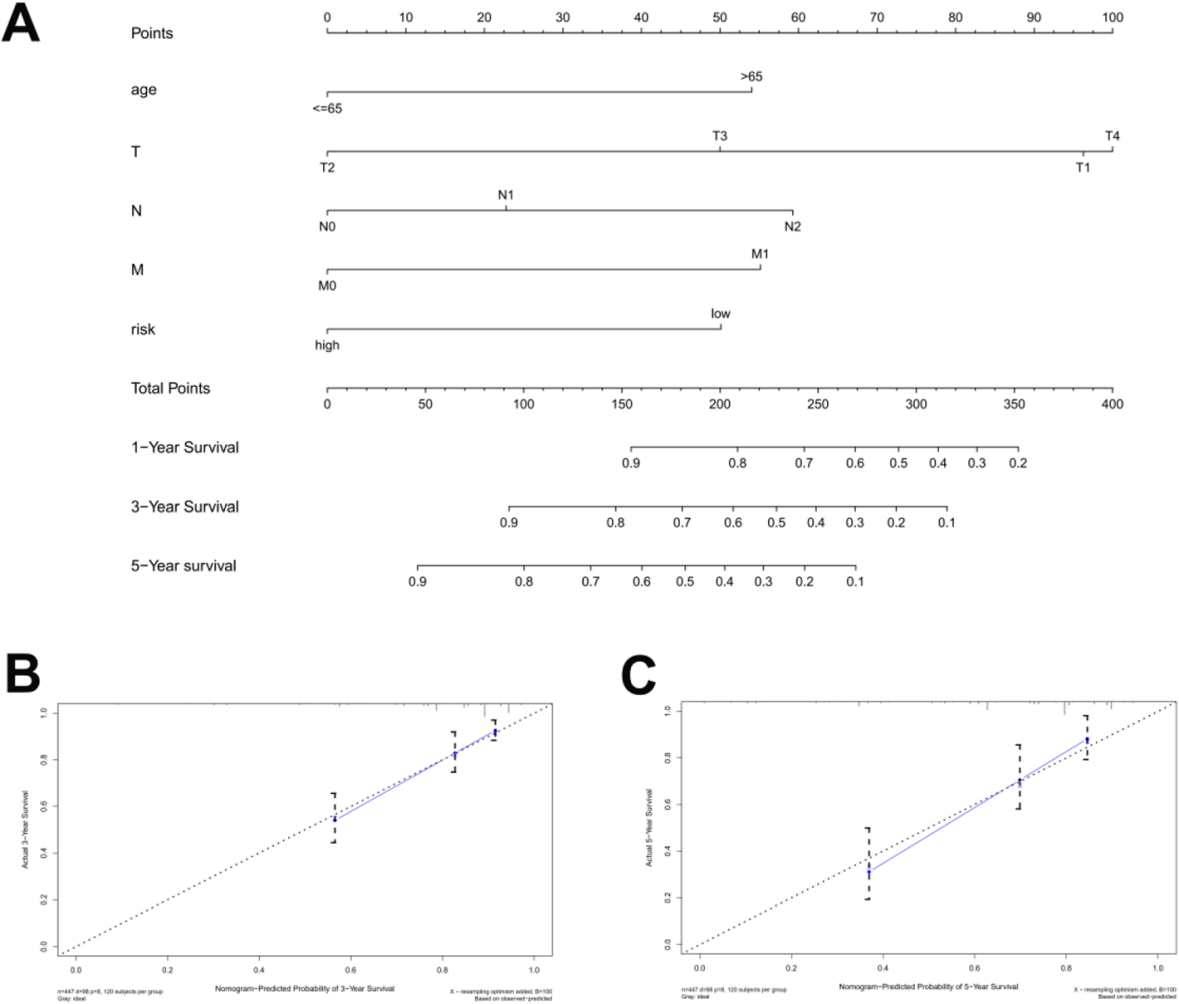
Nomogram model (**a**) to predict 1-, 3-, and 5-year survival rates of colon cancer cases. Calibration graphs indicated that predicted 3- (**b**) and 5- (**c**) year survival rates were close to the actual survival rates

To better understand the influence of these factors on patient survival, a nomogram was drawn to predict 1-, 3-, and 5-year survival rates of colon cancer cases (Fig. 7a). The score obtained from the multivariate analysis was used to predict survival. Accordingly, if a 55-year old colon cancer patient with a tumor of T2N0M0 stage has a high calculated risk score, his or her estimated 5-year survival rate is 40 percent according to the predicted result of nomogram model. Moreover, calibration graphs depicting the differences between nomogram-predicted and actual survival rates of colon cancer patients showed that predicted 3- and 5-year survival rates were close to the actual survival rates (Fig. 7b and c), indicating that this nomogram model accurately predicted survival. Interestingly, the nomogram model was made into a web page at https://doctorwang.shinyapps.io/DynNomapp, which could be easily accessed using desktops, tablets, and smartphones (Additional Figure 2). The prognostic nomogram with an online webserver is more effective for providing accurate and individualized survival prediction in colon cancer patients.

**Fig. 8:**
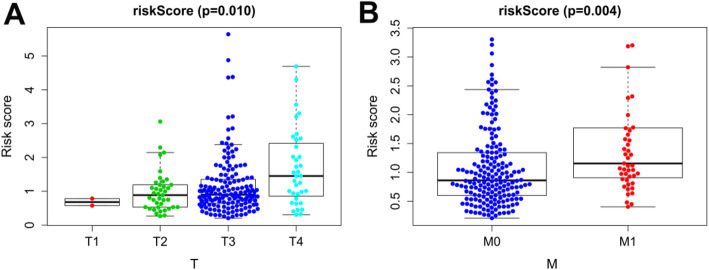
Clinicopathological significance of the prognostic index of colon cancer. *P* values were at different (**a**) tumor infiltration depth and (**b**) distant metastasis

The calculated risk score was associated with other clinicopathological characteristics, including tumor infiltration depth (Fig. 8a) and distant metastasis (Fig. 8b), suggesting that this model may also be predictive of tumor growth and metastasis.

## Discussion

Autophagy has been proved to be associated with multiple types of cancer; however, the relationship between autophagy-related genes and prognosis in colon cancer patients remains largely unknown. To examine levels and patterns of expression of human ARGs in colon cancer, 36 differentially expressed ARGs were identified. GO and KEGG analyses were performed to explore relevant pathways and molecular biological functions. The most significant pathway identified in the KEGG analysis was the p53 signaling pathway. Mutations in the p53 gene occur in most types of malignancies [12], and the p53 signaling pathway plays an important role in cell cycle regulation, metabolism, development and aging, reproduction, and inhibition of tumor formation [13].

To explore the impact of ARGs on prognosis in colon cancer patients, 20 prognosis-related ARGs were identified using univariate Cox regression analysis. Eight of the 20 (40%) ARGs that remained significant after multivariate analysis have been associated with prognosis in colon cancer or other malignant carcinomas. Gil et al. [14] observed that the expression of MAP1LC3C was downregulated in colorectal cancer tissues and was negatively associated with TNM stage. Yuan et al. [15] reported that downregulation of DAPK1 promotes chemoresistance and metastasis of colorectal cancer, while inhibition of DAPK1 promotes the epithelial-to-mesenchymal transition (EMT) of tumor stem cells. Hypermethylation of the MAPK9 promoter region affected the MAPK signaling pathway, focal adhesion, and Wnt signaling pathway in colorectal cancer (CRC) [16]. Soo Jung Lee et al. [17] reported that genetic variation in the TSC1 gene may be useful as a biomarker for predicting patient outcomes after CRC resection surgery. High expression of SERPINA1 has been associated with advanced stage, lymph node metastasis, and poor prognosis of CRC patients, and may be useful as a prognostic marker and candidate therapeutic target for CRC [18]. Salemi et al. [19] observed overexpression of CASP3 in LNCaP and PC-3 prostate cancer cell lines. Upregulation of the ULK3 gene is known to occur in several tumor types, and ULK3 silencing suppresses tumor progression. ULK3 connects two key signaling pathways in the transformation of normal fibroblasts into cancer-associated fibroblasts and, therefore, may represent a potential target for cancer therapy [20]. WIPI1 has been proposed to be a new biomarker related to melanoma at both the gene and protein levels [21]. A previous research has documented that missense mutations constitutively activate oncoproteins [22] and that missense mutations are the most common type of mutation in ARGs associated with colon cancer.

Eight ARGs were used to predict patient prognosis and provide information for individualized treatment. To better understand the utility of clinical features and risk score on predicting outcomes, univariate and multivariate Cox analyses were performed. These analyses revealed that the calculated risk score is an independent predictor of patient prognosis. Because nomograms are widely used prediction tools in oncology, especially for cancer prognosis [23, 24], we developed a nomogram model to visualize the effects of clinical features and risk score on patients’ 3- and 5-year survival probabilities. Calibration graphs verified that the nomogram had high prediction efficiency. Our nomogram of the online version provided more convenient and accurate prediction for colon cancer patients, it could be easily accessed by researchers and clinicians. As mentioned above, our risk score was associated with tumor infiltration depth and distant metastasis, suggesting that it may be related to the development and migration of colon cancer.

This study had several limitations. Subsequent molecular biological experiments are needed to further examine the function of ARGs in colon cancer development and to better understand carcinogenic mechanisms. Additional clinical cases will be required to maximize stability and the predictive ability of our established model.

In conclusion, our analysis of gene expression profiles and corresponding clinical characteristics identified prognosis-related ARGs in colon cancer. Genes associated with autophagy may represent new targets for developing improved treatment options and interventions for patients with colon cancer. Based on the molecular characteristics of autophagy, we constructed a new risk scoring model, which can effectively evaluate the prognosis of colon cancer patients. Additional studies are needed to validate the findings of this study and provide a basis for individualized treatment.

## Supplementary Information

**Additional file 1: Table 1.** Clinicopathologic characteristics of TCGA colon cancer patients.

**Additional file 2: Figure 1.** Flow chart of this study.

**Additional file 3: Figure 2.** Online version of the nomogram model.

## Data Availability

All the data and materials are available.

## Abbreviations

ARG: Autophagy-related gene
CEA: Carcinoembryonic antigen
GO: Gene oncology
KEGG: Kyoto encyclopedia of genes and genomes
ROC: Receiver operator characteristic curve
AUC: Area under curve
LogFC: Log fold change
FDR: False discover rate
CC: Cellular components
BP: Biological processes
MF: Molecular functions
CRC: Colorectal cancer
