# Supplementary material for: Development of prognosis model for colon cancer based on autophagy-related genes
Source: World J Surg Oncol. 2020 Oct 30;18:285. doi: 10.1186/s12957-020-02061-w (PMC7602324; doi:10.1186/s12957-020-02061-w)
Supplement: Supplementary file 1 — Additional file 1: Table 1. Clinicopathologic characteristics of TCGA colon cancer patients. [file 12957_2020_2061_MOESM1_ESM.doc]

**Additional Table 1** Clinicopathologic characteristics of TCGA colon cancer patients

| **Clinicopathologic features** |  | **Total(459)** | **%** |
| --- | --- | --- | --- |
| **age at diagnosis(year)** |  | 68(31-90) |  |
| **sex** | male  female | 243  216 | 52.9  47.1 |
| **race** | asian  black or african american  white | 11  233  213 | 2.5  51.0  46.5 |
| **radiation** | yes  no | 13  446 | 2.8  97.2 |
| **pharmarceutical** | yes | 163 | 35.5 |
|  | no | 296 | 64.5 |
| **stage** | stage I  stage II  stage III  stage IV | 68  166  146  65 | 15.3  37.3  32.8  14.6 |
| **tumor(T)** | T1  T2  T3  T4 | 11  78  313  56 | 2.5  17.0  68.2  12.3 |
| **node (N)** | positive  negative | 189  270 | 41.2  58.8 |
| **metastasis(M)**  **living status** | positive  negative  alive  dead | 67  341  357  102 | 16.4  83.6  77.8  22.2 |
