# Supplementary figures and images for: Development of prognosis model for colon cancer based on autophagy-related genes
Source: World J Surg Oncol. 2020 Oct 30;18:285. doi: 10.1186/s12957-020-02061-w (PMC7602324; doi:10.1186/s12957-020-02061-w)

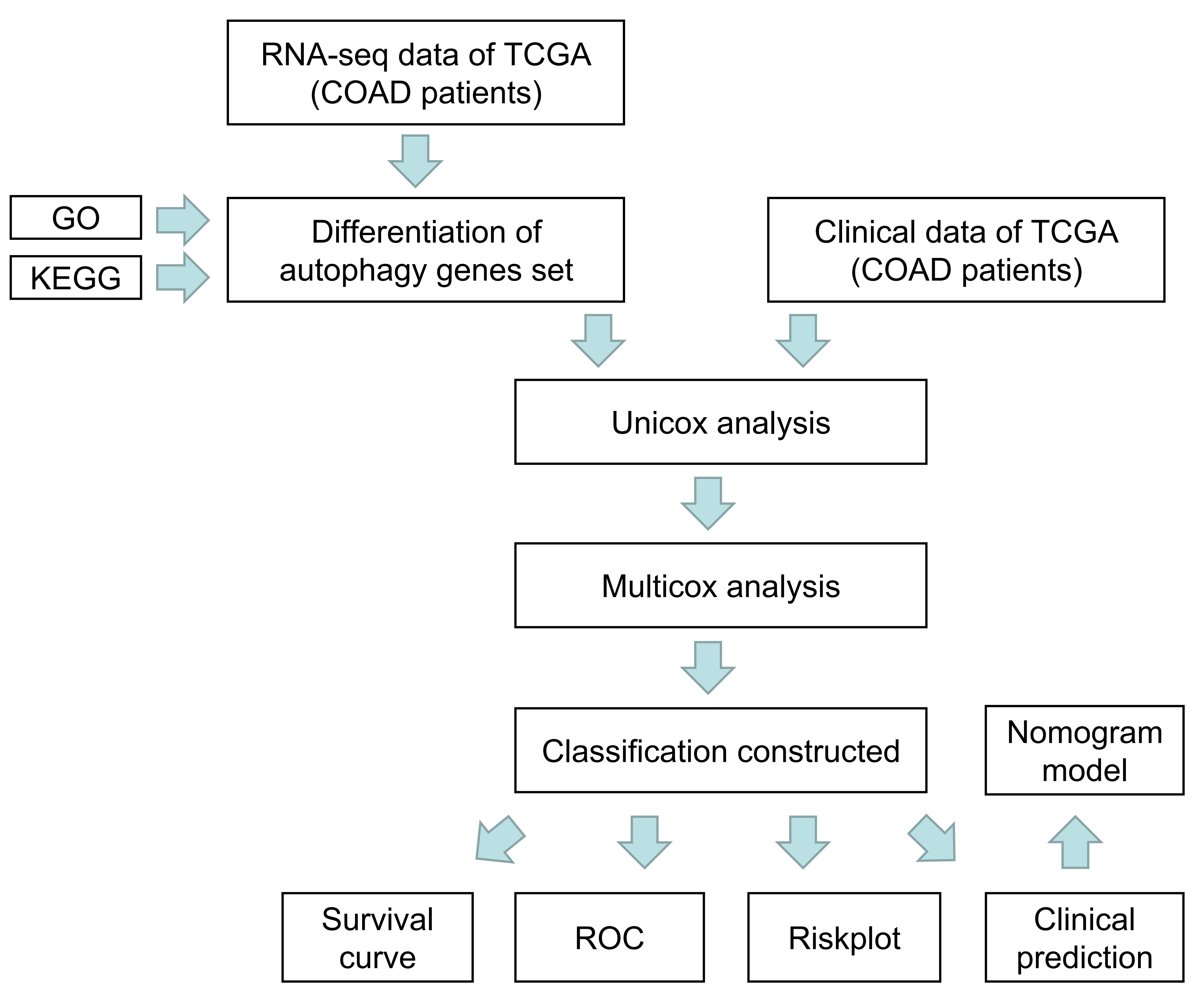

Supplement: Supplementary file 2 — Additional file 2: Figure 1. Flow chart of this study. [file 12957_2020_2061_MOESM2_ESM.tif]

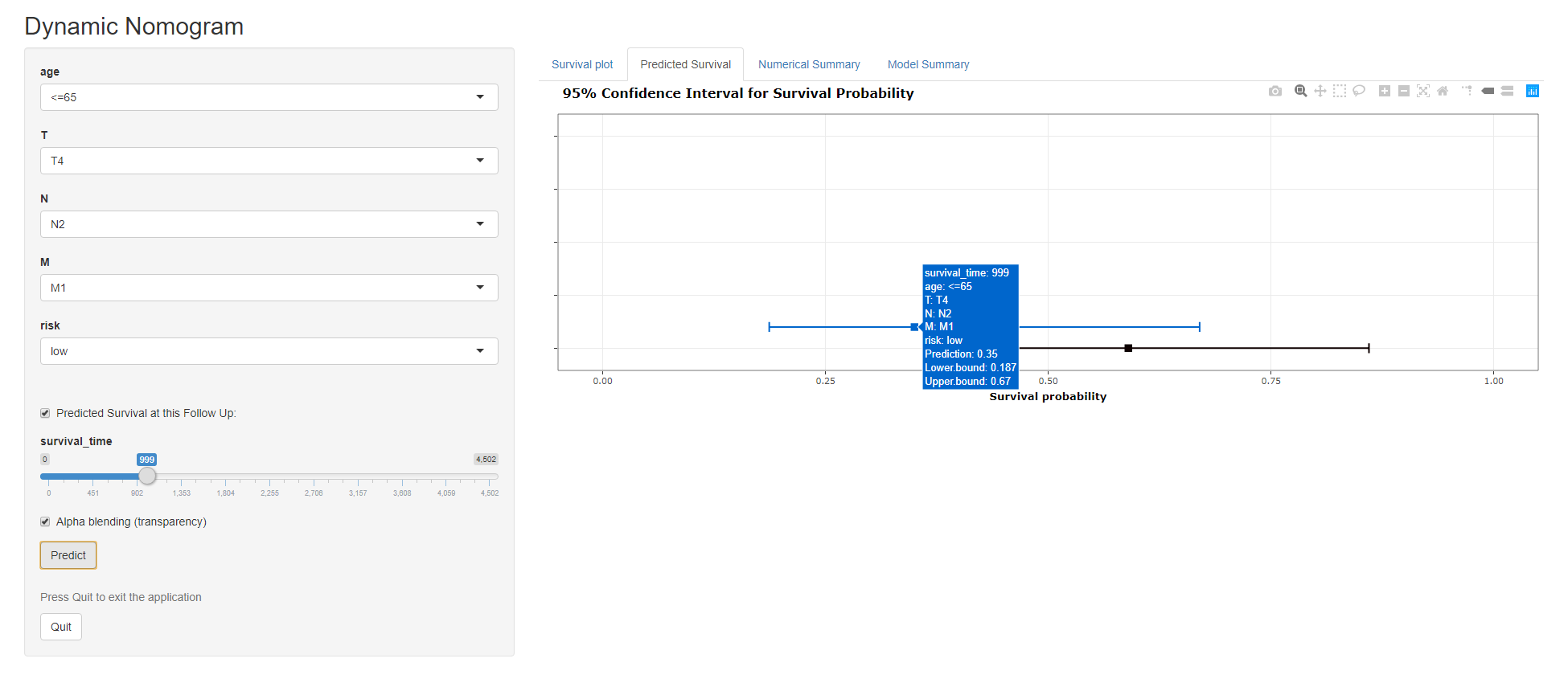

Supplement: Supplementary file 3 — Additional file 3: Figure 2. Online version of the nomogram model. [file 12957_2020_2061_MOESM3_ESM.tif]
